# Loss of calcium‐dependent phospholipase A2 contributes to multi‐omic changes in mouse denervated skeletal muscle

**DOI:** 10.14814/phy2.70906

**Published:** 2026-05-21

**Authors:** Agnieszka Czyżowska‐Froemling, Hongyang Xu, Kylene Harold, Jessica Thomason, Kara Kneuper, Elizabeth Duggan, Atul Pranay, Jie Zhu, Martin‐Paul Agbaga, Constantin Georgescu, Victoria Tyrrell, Valerie O'Donnell, Ken Humphries, Holly Van Remmen, Jacob L. Brown

**Affiliations:** ^1^ Aging and Metabolism Research Program Oklahoma Medical Research Foundation Oklahoma City Oklahoma USA; ^2^ Department of Ophthalmology, and Department of Cell Biology Dean McGee Eye Institute, and University of Oklahoma Health Sciences Center Oklahoma City Oklahoma USA; ^3^ Systems Immunity Research Institute, School of Medicine Cardiff University Cardiff UK; ^4^ Oklahoma City VA Medical Center Oklahoma City Oklahoma USA; ^5^ Department of Health, Nutrition, and Food Sciences Florida State University Tallahassee Florida USA

**Keywords:** atrophy, denervation, lipids, oxylipins, transcriptomics

## Abstract

Age‐related loss of innervation in skeletal muscle is a key driver of sarcopenia. We investigated the role of calcium‐dependent phospholipase A_2_ (cPLA_2_) in denervation‐atrophy. cPLA_2_ mediates the release of polyunsaturated fatty acid substrates, and the oxidation of the free fatty acids generates oxylipins, which are bioactive signaling facilitators. We hypothesized that loss of cPLA_2_ would protect against muscle atrophy by altering hydroperoxide and oxylipin generation, thereby modifying the transcriptome and lipidome of denervated muscle to mitigate atrophy. We used a sciatic nerve transection model in wildtype and cPLA_2_ knockout (KO) mice to test this hypothesis. Surprisingly, oxylipin content was significantly higher in 4,10,11,13,14‐HDoHE, 12‐HEPE, 9,10‐EpOME, and 12,13‐EpOME in gastrocnemius muscle from mice with genetic deletion of cPLA_2_ compared to wildtype controls. We observed reductions in several glycolytic intermediates after denervation such as fructose‐6‐phosphate, glucose‐6‐phosphate, fructose‐1,6‐bisphosphate, and phosphoenol pyruvate. Both alpha‐hydroxy‐glutarate and glucose‐6‐phosphate were lower in muscle from mice lacking cPLA_2_. Transcriptomic analysis showed that G‐protein coupled receptor signaling was differentially expressed when comparing wildtype and cPLA_2_ KO mice. In contrast to the protective effects previously reported with inhibition of cPLA_2_, we found that genetic deletion of cPLA_2_ did not mitigate denervation‐induced muscle atrophy despite having lower hydroperoxide generation in gastrocnemius muscle.

## 1 INTRODUCTION

Age‐related loss of skeletal muscle mass and function (sarcopenia) contributes to poor quality of life and increases the risk of injury and chronic disease (Rizzoli et al., 2013; Wickham et al., 1989). Denervation damage is a key driver of sarcopenia (Campbell et al., 1973; Deschenes et al., 2010; Jang & van Remmen, 2011; Spendiff et al., 2016). In fact, neuromuscular junction disruption and subsequent denervation that occur with aging precede hallmarks of sarcopenia such as muscle loss, mitochondrial pathologies, contractile dysfunction and impaired protein turnover (Deschenes et al., 2010; Larsson & Ansved, 1995; Spendiff et al., 2016). Therefore, understanding the molecular mechanisms underlying denervation‐induced muscle atrophy may help inform the design of interventions to treat sarcopenia.

We have previously shown that mitochondrial hydroperoxide (R‐OOH) generation is higher in both isolated mitochondria and permeabilized muscle fiber bundles from denervated skeletal muscle (Muller et al., 2007). Further, hydroperoxide generation in muscle correlates with the extent of muscle atrophy in various models of neurogenic muscle atrophy (Pharaoh et al., 2020). In subsequent studies, we reported that these hydroperoxides are primarily phospholipids and oxylipins rather than electron transport chain‐generated reactive oxygen species (Brown et al., 2022; Pharaoh et al., 2020). Lipid peroxidation can produce toxic signaling molecules such as 4‐hydroxynonenal (4‐HNE) (Esterbauer et al., 1991; Schneider et al., 2001; Spickett, 2013). 4‐HNE has been shown to trigger skeletal muscle and mitochondrial pathologies (Dalle‐Donne et al., 2003; del Rio et al., 2005; Gęgotek & Skrzydlewska, 2019; Humphries et al., 1998; Long et al., 2009; Schwarzer et al., 2015; Zablocka‐Slowinska et al., 2019). Further, we have shown that scavenging lipid hydroperoxides via pharmacological intervention (Brown et al., 2022; Brown et al., 2025) or overexpression of phospholipid glutathione peroxidase (Xu et al., 2023) mitigates denervation and age‐related muscle pathologies.

**FIGURE 1:**
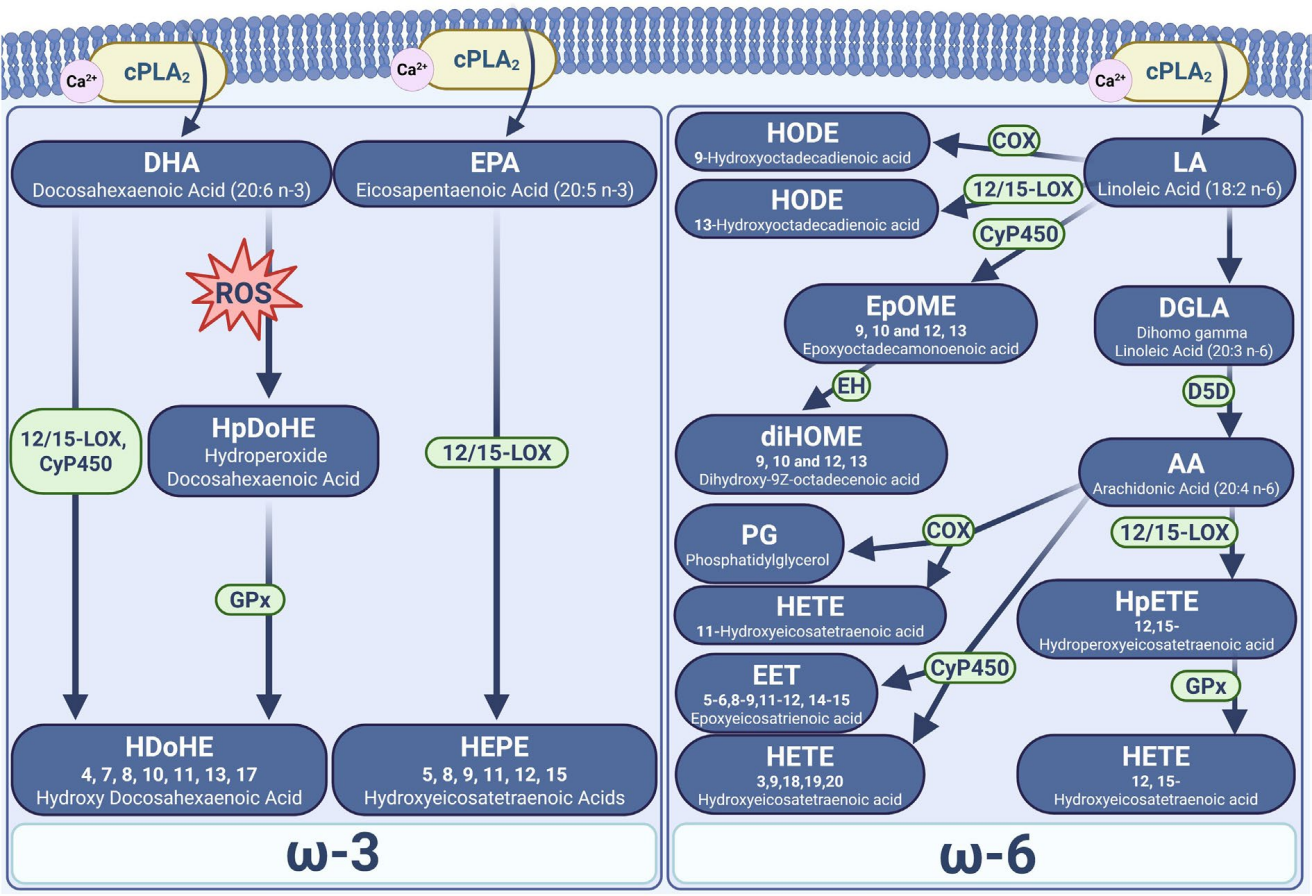
Schematic showing various pathways in which oxylipins are generated. The figure shows various polyunsaturated fatty acid substrates used to generate oxylipins. The figure also shows enzymes that oxidize the polyunsaturated fatty acid substrate to generate an oxylipin.

Omega‐3 and ‐6 polyunsaturated fatty acids (PUFAs), including arachidonic acid (AA), linoleic acid (LA), eicosapentaenoic acid (EPA), docosahexaenoic acid (DHA), and others, which are enriched in the membrane phospholipid bilayer, are a major source of lipid hydroperoxides and oxylipin signaling molecules (Burke & Dennis, 2009). A simplified schematic illustrating the source and generation of oxylipin mediators is illustrated in Figure 1. In response to external or internal stimuli or insult, these fatty acids are released from the phospholipid bilayer by phospholipase (PLA_2_) enzymes to provide substrates for the enzymatic generation of oxylipins by 12/15‐lipoxygenase, cytochrome p450, or cyclooxygenase enzymes [18]. The calcium‐dependent phospholipase A_2_ (cPLA_2_) is ubiquitously expressed and binds Ca^2+^ at its C2 domain, leading to a conformational change which enables membrane binding (Chiba et al., 2004; Ohto et al., 2005; Pickard et al., 1999; Sharp & White, 1993).

The physiological importance of cPLA_2_ has been documented in studies using genetically altered mice lacking the enzyme (Bonventre et al., 1997; Brown et al., 2009). cPLA_2_ knockout (KO) mice were shown to have protective neurological effects following stress and injury in brain and spinal cord (Bonventre et al., 1997; Uozumi et al., 1997). These protective effects include smaller cerebral infarcts, reduced brain edema, less neurologic impairments, and reduced atrophy of dendrites in the spinal cord following injury (Liu et al., 2021). Blocking cPLA_2_ also has protective effects in skeletal muscle. We have previously shown that cPLA_2_ protein content is higher in denervated muscle when compared to innervated muscle, and arachidonoyl trifluoromethyl ketone (AACOCF_3_), a phospholipase inhibitor that reduces the release of fatty acid substrate for the generation of oxylipins by PLA_2_ enzymes, lowers the state 1 hydroperoxide signal in denervated muscle and mitigates muscle atrophy (Pharaoh et al., 2020). In addition, cPLA_2_ KO mice have been reported to have exaggerated hypertrophic responses in striated muscle related to altered stress related IGF‐1 signaling and enhanced growth regulatory signaling pathways that promote muscle growth (Haq et al., 2003). Collectively, these data suggest that blocking oxylipin generation via cPLA_2_ may be an effective strategy to mitigate denervation‐induced muscle atrophy.

Based on our previous report that inhibition of PLA_2_ protects against denervation‐atrophy and data from others showing that deletion of cPLA_2_ leads to muscle growth, in the current study, we asked whether genetic deletion of cPLA_2_ protects against denervation‐induced muscle atrophy. We hypothesized that cPLA_2_ deletion would mitigate muscle atrophy, hydroperoxide generation, and alter the transcriptome and lipidome of denervated muscle. We also predicted that G protein coupled receptor signaling, a known target of oxylipins (Barquissau et al., 2017), would be altered in denervated muscle from cPLA_2_ KO mice when compared to denervated muscle from wildtype mice.

## 2 METHODS

### 2.1 Animals

We conducted all animal experiments in accordance with the guidelines for the care and use of laboratory animals for the Oklahoma Medical Research Foundation (OMRF). The Institutional Animal Care and Use Committees at the OMRF approved the study. Whole body cPLA_2_ KO mice were generated by two independent groups and the initial phenotypes were reported in Nature in 1997 (Bonventre et al., 1997). These mice develop normally, though they exhibit some fertility issues (Bonventre et al., 1997; Brown et al., 2009). cPLA_2_ KO mice were acquired by Dr. Van Remmen from Dr. Joseph Bonventre at the Harvard Stem Cell Institute and bred and maintained on a C57BL/6J genetic background at OMRF. Experimental mice were group housed, kept on a 14:10 h light: dark cycle, and had access to standard rodent chow (LabDiet 5001) and water ad libitum. A combination of male and female mice was used for all experiments in the study. We did not observe any sex differences, so male and female samples were pooled for all experiments. Mice were euthanized by CO2 asphyxiation followed by cervical dislocation.

### 2.2 Body composition measurement

Two days prior to denervation surgery, we used quantitative magnetic resonance (QMR, Echo Medial Systems, Houston, TX, USA) to assess body composition as previously described (Borowik et al., 2024).

### 2.3 Sciatic nerve transection surgeries

We performed sciatic nerve transection on the right hindlimb and sham surgery on the left in 6‐month‐old male and female C57BL/6J mice as previously described (Bhattacharya et al., 2014; Pharaoh et al., 2020). We euthanized mice 7 days after the surgery and collected tissues. After sacrifice, we compared muscle from the following groups: sham wildtype, sham cPLA_2_ KO, denervated wildtype, and denervated cPLA_2_ KO.

### 2.4 Histology

After excision, gastrocnemius muscles were embedded in optimal cutting temperature (O.C.T.) compound (VWR, Radnor, PA, USA) using TissueTek Cryomolds (Sakura, 4557) and snap‐frozen in isopentane pre‐cooled with liquid nitrogen to preserve myofiber alignment. Frozen muscles were cryosectioned through the mid‐belly into 8–10 μm cross‐sections, and then the sections were mounted onto glass slides. Sections were air‐dried at room temperature for approximately 10 min and then fixed with 4% paraformaldehyde (PFA) in PBS for 30 min. Following fixation, sections were washed three times for 10 min each with PBS. Afterwards, sections were incubated in immunobuffer (50 mM glycine, 0.033% saponin, 0.25% bovine serum albumin, 5% serum corresponding to the host species of the secondary antibody, and 0.05% sodium azide) for 2 h at room temperature, as previously described (Xu et al., 2021). Primary antibodies (anti‐dystrophin (Sigma, D8168)) for depicting sarcolemma, and anti‐cPLA2 (Cell Signaling #2832) (1:100) diluted in immunobuffer were then applied, and sections were incubated overnight at room temperature in a humidified, airtight chamber. After primary antibody incubation, sections were washed with immunobuffer and incubated with secondary antibodies (goat anti‐mouse Alexa Fluor 647 and goat anti‐rabbit Alexa Fluor 488; Life Technologies) for 2 h at room temperature in the dark. Sections were then washed twice with PBS for 10 min each and mounted with coverslips using mounting medium (Electron Microscopy Sciences, #17989). Immunofluorescence images were acquired at identical magnification (×20) using a Nikon confocal microscope (Nikon Eclipse). Representative images were downloaded as TIF files directly from Nikon NIS software. Unfortunately, tissue for our denervation experiments was used for other experiments such as lipidomics, metabolomics, RNA and protein extraction. We did not have enough gastrocnemius muscle left for histological assessments.

### 2.5 Lipid extraction and LC/MS/MS oxylipin and phospholipid analysis

We homogenized gastrocnemius muscle and prepared samples for lipid extraction and LC/MS/MS lipidomics analysis as we have previously described (Brown et al., 2022). Briefly, we homogenized tissue samples with ceramic beads in 1 mL antioxidant buffer containing 100 μM diethylenetriaminepentaacetic acid (DTPA) and 100 μM M butylated hydroxytoluene (BHT) in phosphate buffered saline using a Bead Ruptor Elite for 30 s at 6 m/s, under cooled nitrogen gas (4°C). We spiked samples with 12‐HETE‐d8 (2.5 ng), 15‐HETE‐d8 (2.5 ng), 13‐HODE‐d4 (2.3 ng), standards (Cayman Chemical) and 10 ng of PE 15:0–18:1‐d7 (Avanti, Alabaster, Alabama 35,007) prior to homogenization. We then extracted lipids as described in Brown et al. (Brown et al., 2022). For oxylipin analysis, we separated lipids by liquid chromatography (LC) using a gradient of 30%–100% B over 20 min (A: Water: Mob B 95:5% + 0.1% glacial acetic acid, B: Acetonitrile: Methanol – 80:15% + 0.1% glacial acetic acid) on an Eclipse Plus C18 Column (Agilent), and analyzed on a Sciex QTRAP® 7500 LC–MS/MS system, Source conditions: TEM 475°C, IS‐2500, GS1 40, GS2 60, CUR 40 and monitored MRM transitions as detailed in the Table S1. Chromatographic peaks were integrated using Sciex OS 3.3.0 software (Sciex). Peaks were only selected when their intensity exceeded a 5:1 signal to noise ratio with at least 7 data points across the peak. The ratio of analyte peak areas to internal standard was taken and lipids quantified using a standard curve made up and run at the same time as the samples.

For oxPL measurements, lipids were separated by liquid chromatography (LC) using a gradient of 50%–100% B over 10 min, followed by 30 min at 100% B (A: methanol: acetonitrile: water, 1 mM ammonium acetate, 60:20:20. B: methanol, 1 mM ammonium acetate), with a flow rate of 0.2 mL/min on a Luna C18 column (15 cm × 2 mm, 3 μm) (Phenomenex), and analyzed on a Sciex QTRAP® 7500 LC–MS/MS system. Source conditions, negative ion mode: TEM 550°C, IS‐3500, GS1 60, GS2 70, CUR 40. Lipids were detecting using MRM monitoring with the following parent to daughter ion transitions: PE 18:0a_20:4(O) and PC 16:0a_20:4(O) [M − H]‐ 782.6/319.2, PE 18:0p_20:4(O) [M − H]‐ 766.6/319.2, PE 18:1p_20:4(O) [M − H]‐ 764.6/319.2, PE 16:0p_20:4(O) [M − H]‐ 738.6/319.2 and PC 18:0a_20:4(O) [M − H]‐ 810.7/319.2. We monitored PE 15:0–18:1‐d7 as internal standard using precursor to production ion transition of: [M − H]‐ 709.5/288.2. We integrated chromatographic peaks using Analyst 1.7 software (Sciex). The criteria for assigning a peak were signal: noise of at least 5:1 and with at least 7 points across a peak. These MRM transitions gave rise to multiple peaks which were integrated as a group. This method was used since MS/MS data showed these peaks to be a mixture of oxidized PLs (not isolated to HETE‐PL's, but also other oxidized FA containing PL's), however it was not possible to assign individual IDs to each peak. The ratio of analyte peak areas to internal standard was taken and displayed as relative values.

### 2.6 Fatty acids analysis

Gastrocnemius muscles were isolated, snap frozen in liquid nitrogen, and stored at −80°C until analysis. The phospholipid composition of the muscle tissue was analyzed using methods described previously (Hopiavuori et al., 2017; Hopiavuori et al., 2018; Schori et al., 2018). Briefly, the gastrocnemius muscles were homogenized in methanol using Next Advantage Bullet Blender Storm 24 (Troy, NY, USA), and diluted 1:40 with 2‐propanol/methanol/chloroform (4:2:1 v/v/v) containing 20 mM ammonium formate and 1.00 μM Phosphatidylcholine 14:0/14:0 (PC, Avanti Research 850,345), 1.00 μM phosphatidylethanolamine 14:0/14:0 (PE Avanti Research, 850,745), and 0.33 μM phosphatidylserine 14:0/ 14:0 (PS Avanti Research 840,033) as internal standards. Samples were introduced into a triple quadrupole mass spectrometer (TSQ Ultra, Thermo Scientific, Waltham, MA, United States) using a chip‐based nano‐Electron Spray Ionization source (NanoMate, Advion, Ithaca, NY, United States) operating in infusion mode. PC lipids were measured using precursor ion scanning of m/z 184, PE lipids were measured using neutral loss scanning of m/z 141, and PS lipids were measured using neutral loss scanning of m/z 185. Quantification of lipid molecular species was performed using the Lipid Mass Spectrum Analysis (LIMSA, University of Helsinki) software's peak model‐fitting algorithm. Data is represented as the relative percent of each measured species within each class (PC, PE, PS) ± standard deviation.

For fatty acid profiling of muscle tissues, total lipids were extracted from the remaining samples used for lipidomics analyses using the Bligh and Dyer method (Bligh & Dyer, 1959) with modifications (Li et al., 2009). To each lipid extract, 15:0 and 17:0 FAs were added as internal standards. The lipid extracts were subjected to acid hydrolysis in methanol to generate fatty acid methyl esters (FAMEs) (Ford et al., 2008). FAMEs were quantified using a gas chromatograph (6890 N, Agilent Technologies, Foster City, CA, United States) with a flame ionization detector (GC‐FID) (Yu et al., 2012). Data are represented as the relative mole percent of each FA species.

### 2.7 Metabolomic analysis

Targeted metabolomics was performed using liquid chromatography–mass spectrometry assays (LC–MS), as previously described (Harold et al., 2024; Harold et al., 2025). Briefly, frozen muscle tissue was pulverized using a Qiagen Tissue Lyser II containing pre‐chilled metal beads, followed by metabolite extraction with methanol: water (8:2 v/v), and sonication & centrifugation. 5 μL of adonitol (0.2 mg/mL, Sigma, A5502) was added as an internal standard to the samples. Metabolite extracts were passed through Captiva EMR‐lipid cartridges for removal of lipids. For quality control (QC), 35 μL from each sample was pooled to generate a QC sample. Supernatant was dried and reconstituted in 100 μL of acetonitrile and water (80:20 v/v) & transferred to glass inserts for LC–MS analysis.

LC–MS analysis was performed on an Agilent 6546 LC/Q‐TOF coupled to an Agilent 1290 Infinity II LC. Liquid chromatographic separation was performed on an InfinityLab Poroshell 120 HILIC‐Z column (Agilent Technologies, Santa Clara, CA) using aqueous ammonium acetate and acetonitrile as mobile phases. Hydrophilic interaction liquid chromatography (HILIC) was used with an injection volume of 1 μL. Data was acquired in a negative ESI full MS scan mode (scan range: m/z 40–1000) using Agilent MassHunter Workstation LC/MS Data Acquisition software version 10.1. Data analysis and peak integration for target metabolites was performed with Agilent MassHunter Quantitative analysis software (version 10.1). Targeted metabolomic profiling focused on glycolytic intermediates, TCA cycle intermediates and amino acids. To identify these metabolites, a custom Agilent Personal Compound Database and Library (PCDL) was created from the Agilent METLIN PCDL. An analytical standard mix of glycolytic & TCA cycle intermediates, and amino acids standard mix samples were also analyzed along with muscle samples for identification of these metabolites in the samples. Relative abundance values for target metabolites were obtained by normalizing the raw data with tissue weights and adonitol (added as an internal standard) values.

### 2.8 Muscle fiber permeabilization, oxygen consumption, and hydroperoxide production rate measurement

Muscle fiber bundles were permeabilized with saponin for the measurement of oxygen consumption and hydroperoxide production using Amplex UltraRed (Invitrogen A36006) and the Oroboros Oxygraph‐2 k (O2k, OROBOROS Instruments, Innsbruck, Austria) with a fluorometer as previously described (Brown et al., 2017; Brown et al., 2020; Pharaoh et al., 2020). We normalized respiration measurements to Antimycin A (Sigma, A8674) to account for non‐mitochondrial oxygen consumption, and OCR and rates of hydroperoxide generation were normalized by milligrams of muscle bundle wet weights weighed on the Acculab AL‐104 scale.

### 2.9 RNA isolation and RNA sequencing

We extracted total RNA from gastrocnemius using TRI reagent solution (Invitrogen, Carlsbad, CA, United States) as previously described (Brown et al., 2017; Brown et al., 2018). RNA sequencing (RNA Seq) was performed at the OMRF Clinical Genomics Core and analyzed by the Geroinformarics Core in the Oklahoma Nathan Shock Center of Excellence in the Biology of Aging as previously described (Qaisar et al., 2020).

Sequencing was performed on an Illumina NovaSeq 6000 instrument with paired‐end 150 bp reads. Sequence reads were trimmed to remove possible adapter sequences and nucleotides with poor quality using Trimmomatic v.0.36. The trimmed reads were mapped to the Mus musculus GRCm39 (mm39) reference genome available on ENSEMBL using the STAR aligner v.2.5.2b. Unique gene hit counts were calculated by using feature Counts from the Subread package v.1.5.2.

We used R packages limma‐voom and edgeR as parts of a two‐stage pipeline for performing Differential Expression (DE) analysis of RNA‐seq data. In short, the edgeR package cleans and organizes the count data, the limma package performs robust statistical testing, while the voom function translates between the two packages. Read‐count normalization and differentially expressed analyses were performed using the edgeR package from Bioconductor. Expression values quantile normalized with the voom function were analyzed for differential expression using the standard functions of the limma package. Moderated *t*‐test *p*‐values were adjusted for multiple testing using the false discovery rate (FDR) method. The moderated *t*‐statistic computed by the Empirical Bayes function in the R package limma (Linear Models for Microarray Data) is a statistically enhanced version of the ordinary *t*‐statistic designed to increase the power and stability of differential expression analysis with small sample sizes. The test is moderated because it uses an Empirical Bayes approach to stabilize the gene‐wise variance estimates. Instead of relying solely on the possibly unreliable variance calculated from the small sample size of a single gene, Empirical Bayes borrows information across the thousands of other genes in the dataset, effectively shrinking each gene's estimated variance toward a common, pooled prior variance. This stabilization prevents extreme variance estimates from leading to spurious significant or non‐significant results, thereby yielding a more powerful and reliable test statistic than the ordinary *t*‐statistic. FDR (*q*.value) < 0.05 and absolute log2 fold change above 1 were used as criteria to filter significantly differentiated genes.

Gene Set Enrichment Analysis (GSEA) was conducted using specialized Bioconductor packages, including fgsea, ReactomePA, and viewPathway, to identify functionally related gene sets (e.g., GO terms, KEGG and Reactome pathways) that were significantly overrepresented among the differentially expressed genes.

Ingenuity Pathway Analysis (IPA, QIAGEN, Redwood City CA, https://www.qiagenbioinformatics.com/products/ingenuitypathway‐analysis) was used to explore significant gene networks and pathways interactively.

### 2.10 Western blot analysis

We performed western blots as previously described (Brown et al., 2018; Brown et al., 2020). Briefly, we homogenized gastrocnemius muscle in RIPA buffer. Protein concentrations were determined using a Bradford assay. We resolved 20–40 μg of protein by sodium dodecyl sulfate‐polyacrylamide gel electrophoresis, transferred to a nitrocellulose membrane (Thermo Scientific Catalog 88,018) and blocked in 5% weight by volume bovine serum albumin (Sigma‐Aldrich A3311) in Tris‐buffered saline with 0.2% Tween 20. We probed membranes overnight for antibodies specific to cPLA_2_ (abcam, ab58375), iPLA_2_ (abcam, ab259950), 15‐LOX (abcam, ab244205), and GPx4 (Santa Cruz, sc‐166,570). We used anti‐rabbit HRP‐conjugated secondary antibody (Cell signaling, 7074) per manufacturer protocols. We used Licor premium Chemiluminescent substrate (Licor, 926–95,000) to develop the western blots. Imaging was performed with G:BOX imaging system (Syngene) and quantified using Alpha View: Protein Simple analysis software.

### 2.11 Statistical analysis

For animal experiments, independent factors were Surgery (Sham or Denervated) and genotype (wildtype or cPLA_2_ KO). A Two‐Way ANOVA was employed for each dependent variable for animal experiments. Where we found significant *F*‐ratios, we used Tukey Kramer post hoc test to determine differences among means. For all experiments, the comparison‐wise error rate, *α*, was set at 0.05 for all statistical tests. We analyzed data and compiled figures using GraphPad Prism (La Jolla, CA, USA) and data expressed as mean ± SEM.

## 3 RESULTS

### 3.1 Characterization of cPLA_2_ KO mice

**FIGURE 2:**
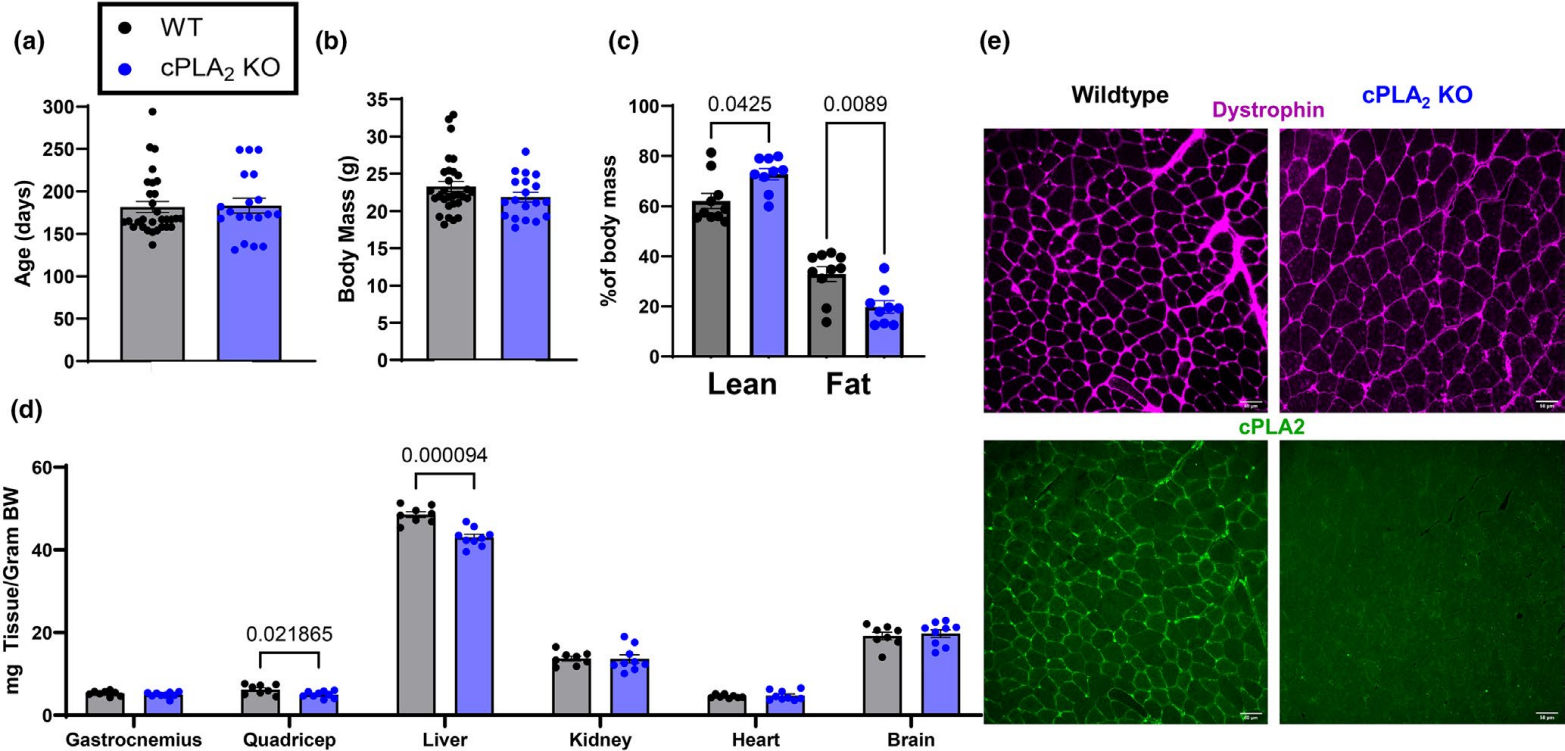
Characterization of cPLA_2_ KO mouse. (a) Age of wildtype and cPLA_2_ KO mice used for the study. (b) Bodymass of wildtype and cPLA_2_ KO mice. (c) Body composition of wildtype and cPLA_2_ KO mice. (d) Tissue masses of various organs normalized to body mass from wildtype and cPLA_2_ KO mice. (e) Representative dystrophin staining images from TA muscle in wildtype and cPLA_2_ KO mice to show the outline of the muscle fibers. Scale bar in the images is 50 μm. An *n* = 8–19 per group was used.

Figure 2 illustrates the basic characteristics of the mice used in this study. Age and body weight did not differ between groups (Figure 2a,b). While mass was unchanged, body composition was altered by genotype. The percentage lean mass was ~20% higher in cPLA_2_ KO mice when compared to wildtype mice, and the percentage of fat mass was ~50% lower in cPLA_2_ KO mice when compared to wildtype mice (Figure 2c). Quadricep mass and liver mass expressed relative to body mass were lower in cPLA_2_ KO mice when compared to wildtype mice (Figure 2d). Gastrocnemius, heart, brain, and kidney mass were not different between groups (Figure 2d). Figure 2e shows that the protein content of cPLA_2_ is localized on the sarcolemma in wildtype mice and cPLA_2_ is undetectable in KO mice.

### 3.2 cPLA_2_ , iPLA2, 12/15‐lox, and GPx4 protein content in sham and denervated muscle from wildtype and cPLA_2_ KO mice

**FIGURE 3:**
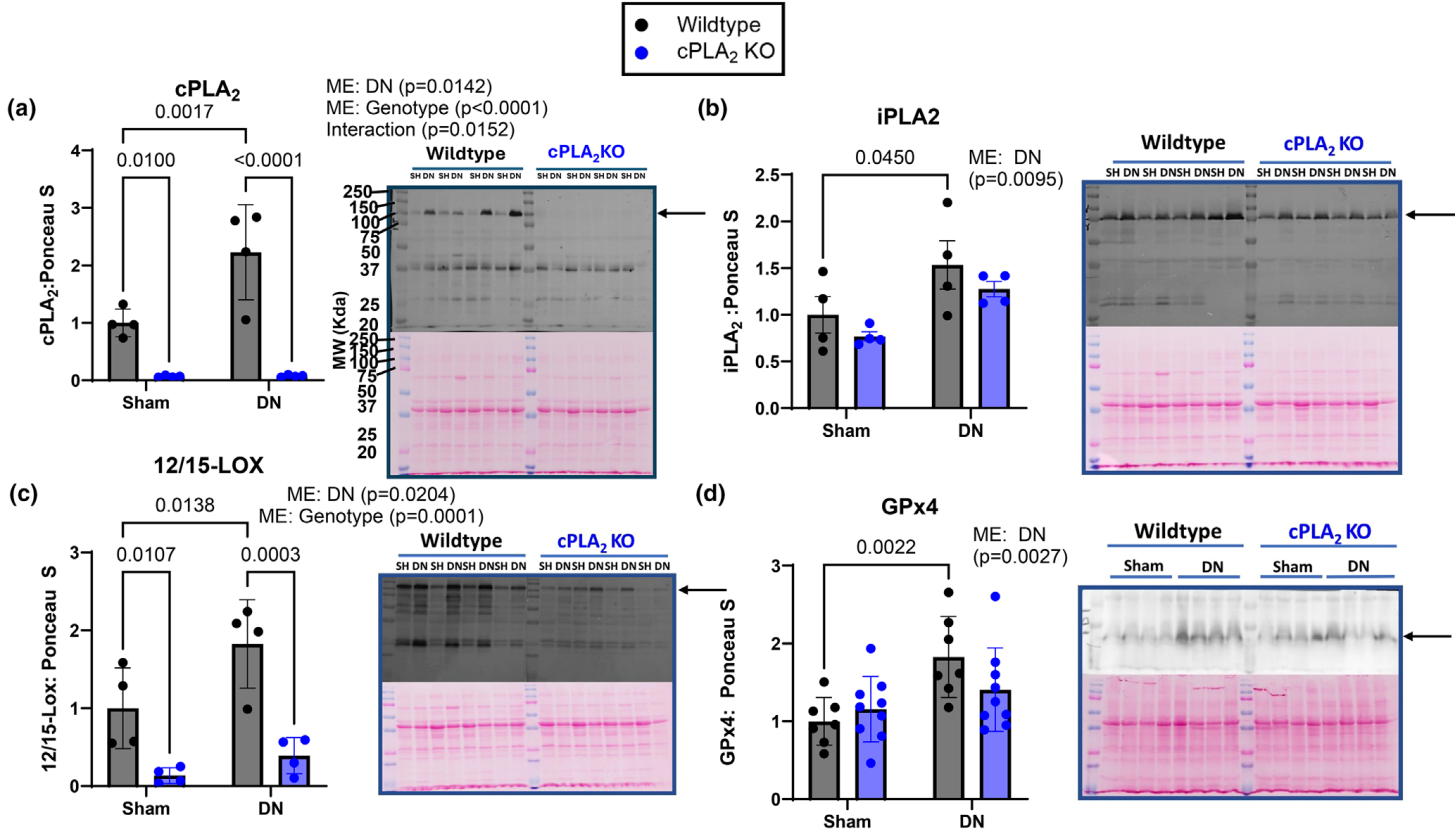
CPLA_2_, iPLA2, 12/15‐Lox, and GPx4 protein content in sham and denervated muscle from wildtype and cPLA_2_ KO mice. (a) cPLA_2_ protein content in sham and denervated gastrocnemius muscle from wildtype and cPLA_2_ KO mice. (b) iPLA_2_ protein content in sham and denervated gastrocnemius muscle from wildtype and cPLA_2_ KO mice. (c) 12/15‐Lox protein content in sham and denervated gastrocnemius muscle from wildtype and cPLA_2_ KO mice. (d) GPx4 protein content in sham and denervated gastrocnemius muscle from wildtype and cPLA_2_ KO mice. An *n* = 4–9 per group was used. ME: Main Effect.

PLA_2_ enzymes release substrates such as arachidonic acid to be oxygenated by enzymes such as 12/15‐Lipoxygenase (12/15‐LOX) to generate oxylipins (mono‐oxygenated and lipid hydroperoxides) [18]. cPLA_2_ protein content in hindlimb skeletal muscle was 2‐fold higher in denervated compared to sham control muscle in wildtype mice (Figure 3a). As expected, cPLA_2_ protein content was not detected in sham or denervated muscle from cPLA_2_ KO mice (Figure 3a). The protein content of iPLA_2_ was ~50% higher in denervated muscle compared to sham, but not different between genotypes (Figure 3b), confirming the specificity of the gene deletion for the cPLA_2_ isoform of PLA_2_. 12/15‐LOX expression was ~2‐fold higher in denervated muscle when compared to sham (Figure 3c). The level of 12/15‐LOX, the enzyme responsible for converting fatty acids to oxylipins such as 12‐ and 15‐HETE, 17‐HDOHE, and 13‐HODE, was significantly lower (~80%) in cPLA_2_ KO mice versus wildtype mice (Figure 3c). We also measured the level of Phospholipid Glutathione Peroxidase (GPx4), the enzyme that reduces lipid hydroperoxides into mono‐oxygenated oxylipins (Forcina & Dixon, 2019; Imai & Nakagawa, 2003; Seibt et al., 2019). GPx4 protein content was higher in denervated muscle when compared to sham muscle from wildtype mice, but not different in sham or denervated muscle from cPLA_2_ KO mice (Figure 3d).

### 3.3 Deletion of cPLA_2_ does not mitigate denervation‐induced muscle atrophy

**FIGURE 4:**
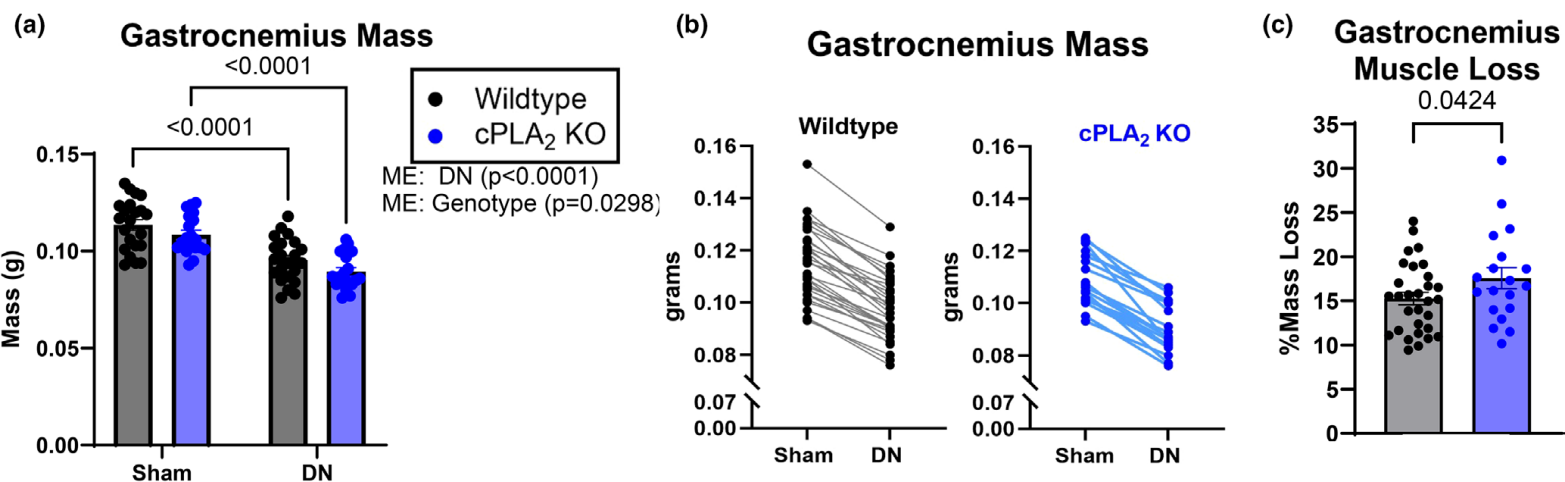
WT and cPLA_2_ KO mice response to denervation. (a) Gastrocnemius mass normalized to bodyweight from sham and denervated muscle in wildtype and cPLA_2_ KO mice. (b) Matched mass loss from sham and denervated muscle in wildtype and cPLA_2_ KO mice. (c) Percent mass loss in denervated gastrocnemius muscle from wildtype and cPLA_2_ KO mice. An *n* = 13–22 per group was used. ME, main effect.

Our previous study using a cPLA_2_ inhibitor, AACOCF_3_, showed protection against denervation induced muscle atrophy (Pharaoh et al., 2020). Here, under similar conditions (7‐days post denervation) we measured muscle mass in wildtype and cPLA_2_ KO mice. As expected, denervation induced loss of muscle mass in both wildtype and cPLA_2_ KO mice (Figure 4a,b). However, contrary to our previous results using a cPLA_2_ pharmacologic inhibitor AACOCF_3_, denervation in the cPLA_2_ KO mice resulted in ~15% greater relative muscle atrophy in cPLA_2_ KO mice compared to wildtype mice (expressed as the percentage of gastrocnemius mass lost comparing the sham and denervated limb in each individual mouse, Figure 4c).

### 3.4 Denervation lowers most glycolytic intermediates, and deletion of cPLA2 lowers D‐glucose‐6‐phosphate in muscle

**FIGURE 5:**
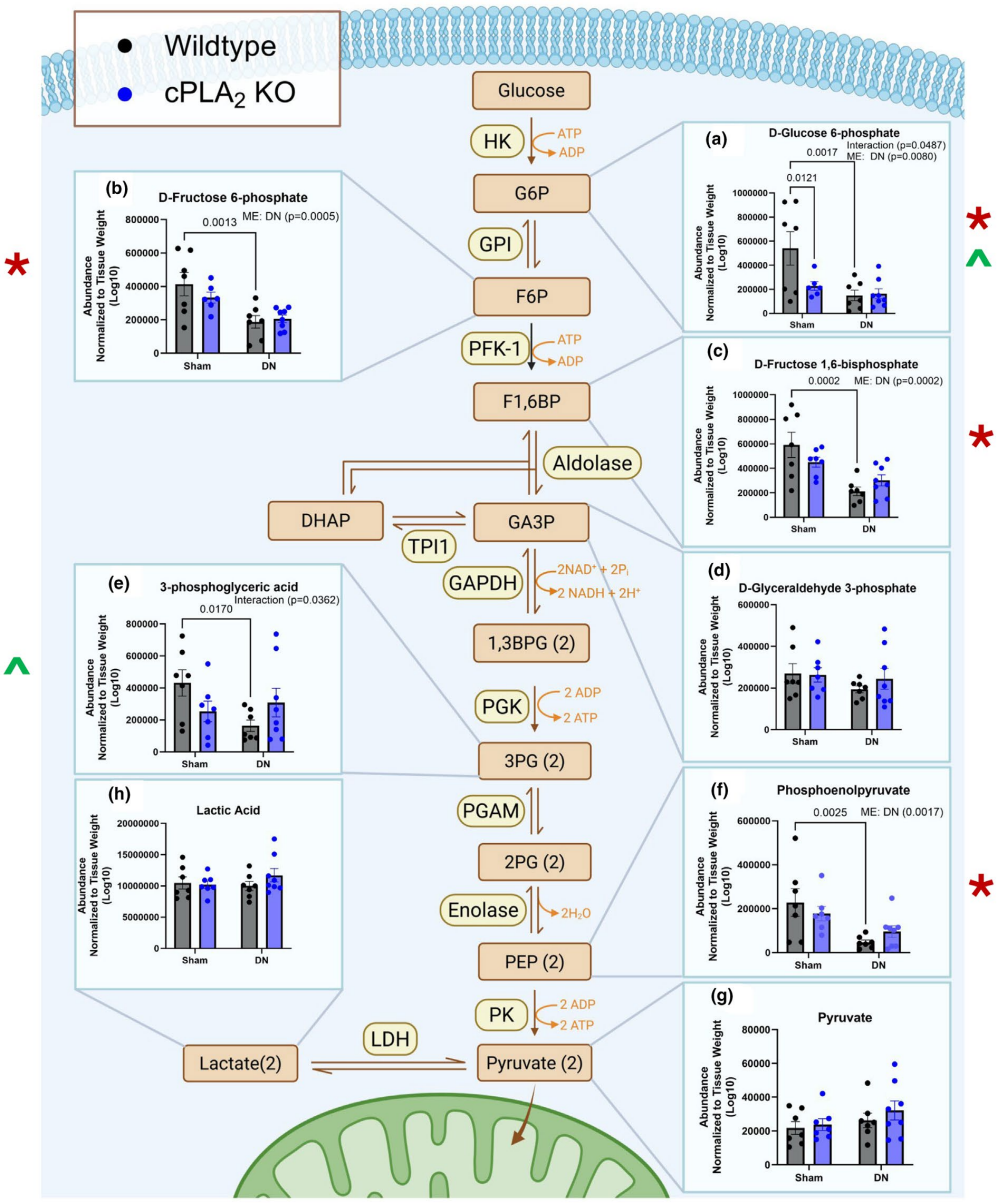
Denervation lowers glycolysis metabolites, and deletion of cPLA2 lowers D‐glucose‐6‐phosphate in muscle. (a) D‐Glucose‐6‐Phosphate normalized to gastrocnemius weight. (b) D‐Fructose‐6‐Phosphate normalized to gastrocnemius weight. (c) D‐Fructose‐1,6‐BisPhosphate normalized to gastrocnemius weight. (d) D‐Glyceraldehyde‐3‐Phosphate normalized to gastrocnemius weight. (e) 3Phosphoglyceric Acid normalized to gastrocnemius weight. (f) Phosphoenolpyruvate normalized to gastrocnemius weight. (g) Pyruvate normalized to gastrocnemius weight. An *n* = 6–8 per group was used. *denotes a main effect difference with denervation; *denotes a main effect difference with denervation; ^denotes an interaction between sham and denervated WT and cPLA_2_ KO.

Prior work shows that denervation (Kostrominova, 2022) and PLA_2_ enzymes (Prunonosa Cervera et al., 2021) alter muscle metabolism. D‐Glucose 6‐phosphate was ~75% lower in DN muscle and muscle from cPLA_2_ KO mice when compared to sham or wildtype controls (Figure 5a). D‐Fructose 6‐phosphate and D‐Fructose 1,6‐bisphosphate were ~50% lower in DN muscle when compared to sham (Figure 5b,c). D‐Glyceraldehyde 3‐phosphate was not different between groups (Figure 5d). 3‐Phosphoglyceric acid and Phosphoenolpyruvate were ~65% lower in DN muscle when compared to sham control (Figure 5e,f). Pyruvate and lactic acid were not different between groups.

### 3.5 Deletion of cPLA_2_ lowers muscle DL‐α‐hydroxyglutaric acid content and hydroperoxide generation in denervated muscle

**FIGURE 6:**
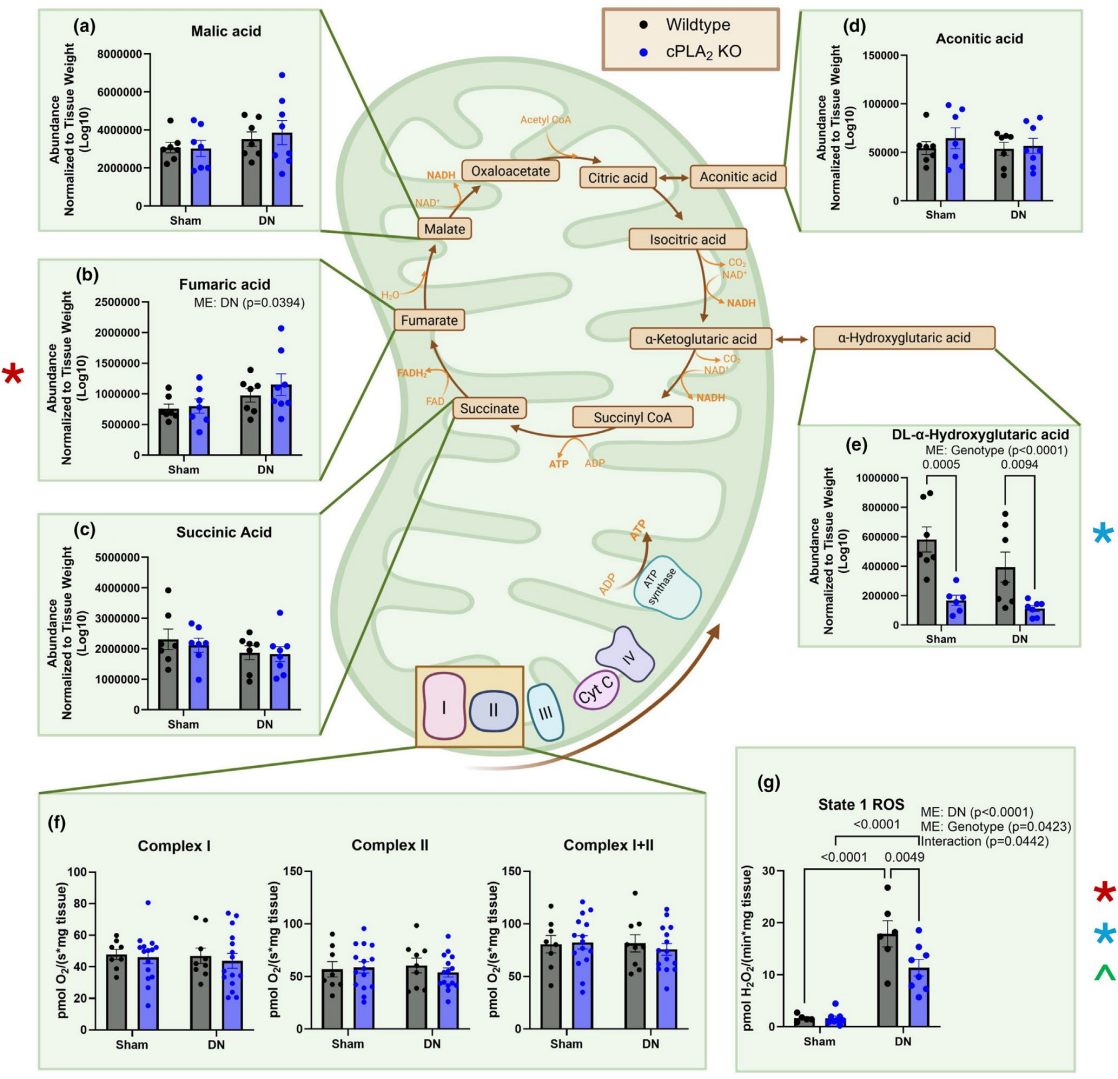
Deletion of cPLA_2_ lowers muscle DL‐α‐hydroxyglutaric acid content and hydroperoxide generation in denervated muscle. (a) Malic Acid normalized to gastrocnemius weight. (b) Fumaric Acid normalized to gastrocnemius weight. (c) Succinic Acid normalized to gastrocnemius weight. (d) Aconitic Acid normalized to gastrocnemius weight. (e) DL‐Alpha‐hydroxyglutaric Acid normalized to gastrocnemius weight. (f) Complex I, Complex II, and Complex I + II Respiration in permeabilized fiber bundles. (g) State 1 ROS generation in permeabilized fiber bundles. An *n* = 6–15 per group was used. ME: Main Effect. *denotes a main effect difference with denervation; *denotes a main effect difference with genotype; ^denotes an interaction between sham and denervated WT and cPLA_2_ KO.

The TCA metabolites malic acid, fumaric acid, succinic acid, and aconitic acid were not different between groups (Figure 6a–d). However, DL‐α‐hydroxyglutaric acid was ~70% lower in muscle from cPLA_2_ KO mice when compared to muscle from wildtype mice (Figure 6e). Mitochondrial respiration and hydroperoxide generation were measured in permeabilized fiber bundles from red gastrocnemius muscle from sham and denervated wildtype and cPLA_2_ KO mice. Complex I, complex I + II, and complex II respiration were not different between groups (Figure 6f). Hydroperoxide generation was significantly elevated by ~10‐fold in both wildtype and cPLA_2_ KO denervated muscle when compared to sham (Figure 6g). However, hydroperoxide generation was lower in denervated muscle from cPLA_2_ KO mice when compared to wildtype mice (Figure 6g). Collectively, these data indicate that cPLA_2_ deletion significantly attenuates DL‐α‐hydroxyglutaric acid accumulation and mitigates hydroperoxide generation in denervated muscle, despite having no impact on mitochondrial respiratory kinetics or on standard TCA cycle intermediates.

### 3.6 cPLA_2_ deletion alters the muscle polyunsaturated fatty acids

**FIGURE 7:**
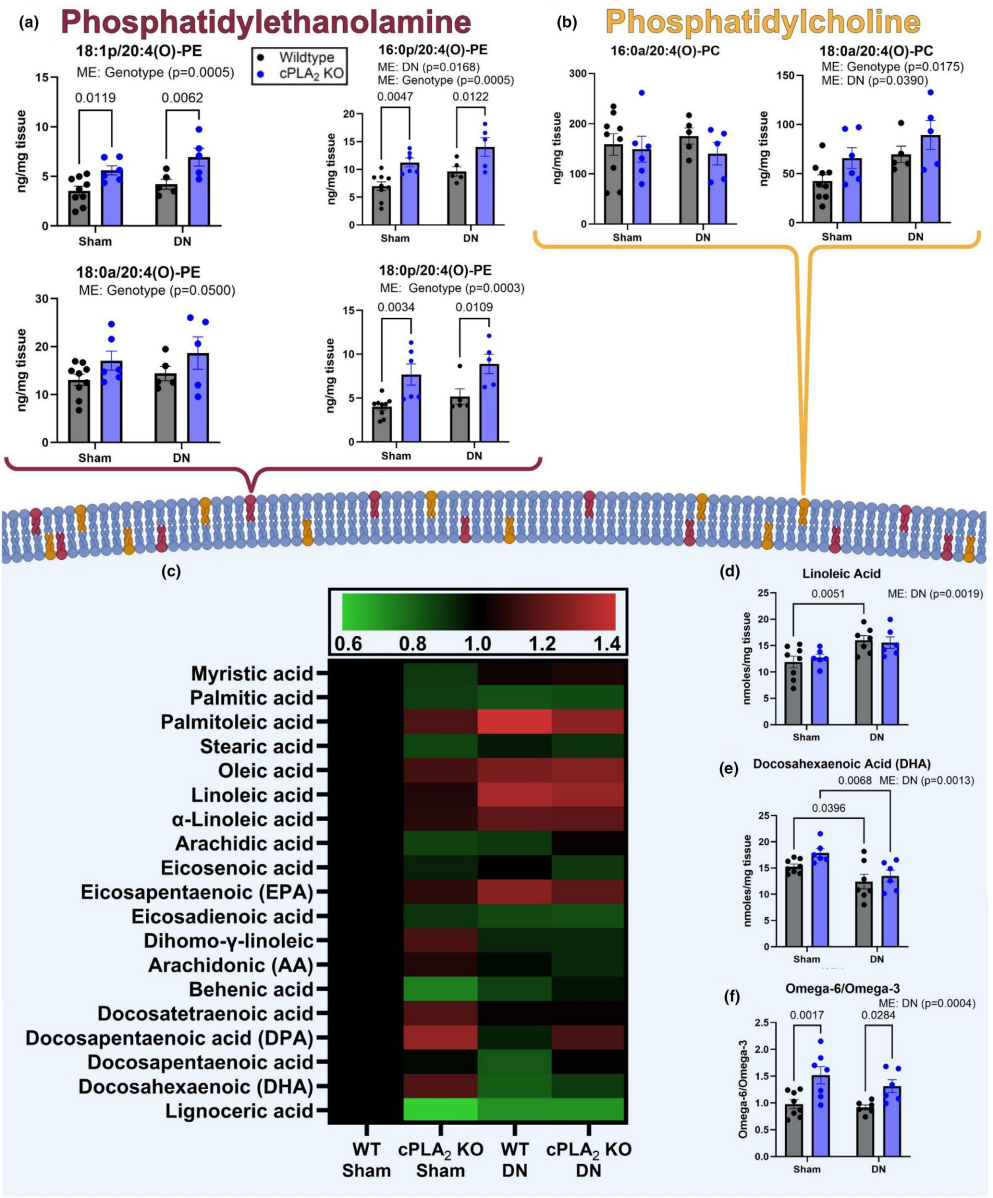
CPLA_2_ deletion alters the muscle polyunsaturated fatty acids. (a) 18:1p20:1(o)‐PE phospholipid content. 16:0p 20:4(O)‐PE phospholipid content. 18:0a20:4(O)‐PE phospholipid content. 18:0p20:4(O)‐PE phospholipid content. (b) 16:0a20:4(O)‐PC phospholipid content. 18:0a20:4(O)‐PC phospholipid content. (c) Relative content of PUFA substrate. (d) Linoleic Acid content. (e) DHA Content. (f) Omega 6/3 ratio. An *n* = 5–9 per group was used. ME, main effect.

Genetic deletion of cPLA_2_ would be predicted to reduce or limit the release of PUFAs from the membrane phospholipid pool, in particular release of arachidonic acid, to serve as substrates for the generation of oxylipins. To investigate the impact of cPLA_2_ deletion on fatty acid levels and oxylipin generation, we measured oxidized phospholipids, PUFA substrate, and oxylipin levels in gastrocnemius muscle from sham and denervated wildtype and cPLA_2_ KO mice. Levels of oxidized phospholipids are shown in Figure 7a,b. As expected, the levels of 18:0p/20:4(O)‐PE (Figure 7a), 16:0p/20:4(O)‐PE (Figure 7a), 18:0a/20:4(O)‐PE (Figure 7a), 18:0p/20:4(O)‐PE (Figure 7a), and 18:0a/20:4(O)‐PC (Figure 7b) were 10%–20% higher in denervated muscle from cPLA_2_ KO mice compared to sham muscle from wildtype mice, demonstrating lack of release of arachidonic acid, 20:4n6 from the membranes of cPLA_2_ KO mice. 16:0a/20:4 (O)‐PC was not changed in DN or cPLA_2_ KO mice compared to control (Figure 7b). Figure 7c shows the levels of specific fatty acids measured in muscle from sham and denervated wildtype and cPLA_2_ KO mice. Linoleic acid and DHA levels were the only fatty acids to show a change in denervation, and there were no changes in response to the loss of cPLA_2_ (Figure 7d,e). Linoleic acid levels were ~30% higher in wildtype denervated muscle compared to wildtype sham muscle (Figure 7d), while DHA was ~20% lower in denervated muscle from cPLA_2_ KO mice when compared to sham muscle from cPLA_2_ KO mice (Figure 7e). The omega 6:3 ratio was ~35% higher in cPLA_2_ KO denervated muscle compared to wildtype sham muscle (Figure 7f), but not altered in the cPLA_2_ KO mice. Thus, contrary to the prediction that cPLA_2_ deletion would limit substrate supply, the levels of PUFAs and arachidonic acid‐containing oxidized phospholipids were largely preserved, or even elevated, in cPLA_2_ KO muscle, pointing to alternative mechanisms for substrate maintenance.

### 3.7 cPLA_2_ deletion alters the muscle oxylipin profile

**FIGURE 8:**
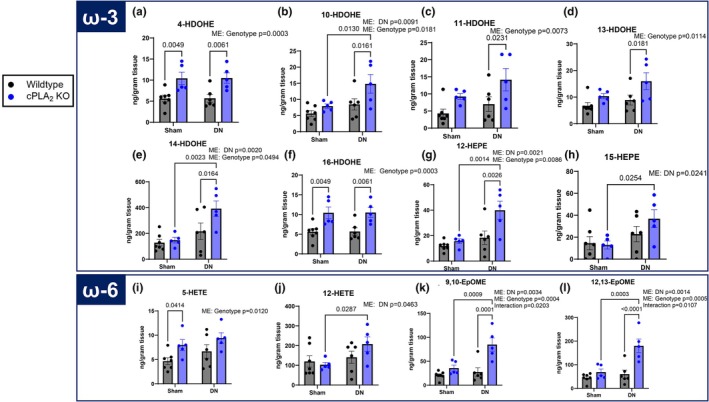
CPLA_2_ deletion alters the muscle oxylipin profile. (a) 4‐HDoHE content in sham and denervated gastrocnemius muscle from wildtype and cPLA_2_ KO mice. (b) 10‐HDoHE content in sham and denervated gastrocnemius muscle from wildtype and cPLA_2_ KO mice. (c) 11‐HDoHE content in sham and denervated gastrocnemius muscle from wildtype and cPLA_2_ KO mice. (d) 13‐HDoHE content in sham and denervated gastrocnemius muscle from wildtype and cPLA_2_ KO mice. (e) 14‐HDoHE content in sham and denervated gastrocnemius muscle from wildtype and cPLA_2_ KO mice. (f) 16‐HDoHE content in sham and denervated gastrocnemius muscle from wildtype and cPLA_2_ KO mice. (g) 12‐HEPE content in sham and denervated gastrocnemius muscle from wildtype and cPLA_2_ KO mice. (h) 15‐HEPE content in sham and denervated gastrocnemius muscle from wildtype and cPLA_2_ KO mice. (i) 5‐HETE content in sham and denervated gastrocnemius muscle from wildtype and cPLA_2_ KO mice. (j) 12‐HETE content in sham and denervated gastrocnemius muscle from wildtype and cPLA_2_ KO mice. (k) 9,10‐EpOME content in sham and denervated gastrocnemius muscle from wildtype and cPLA_2_ KO mice. (l) 12,13‐EpOME content in sham and denervated gastrocnemius muscle from wildtype and cPLA_2_ KO mice. An *n* = 5–7 per group was used. ME, main effect.

Loss of cPLA_2_ would be expected to alter the availability of PUFA substrate for oxylipin generation. We measured the effects of genotype and denervation on the oxylipin profile. With respect to levels of oxylipins derived from ω‐3 fatty acids, we found that 4‐HDOHE was ~40% higher in denervated muscle when compared to sham (Figure 8a). Meanwhile, the oxylipins 10‐HDoHE, 11‐HDoHE, 13‐HDoHE, 14‐HDoHE, 16‐HDoHE, and 12‐HEPE were higher in denervated cPLA2 KO muscle when compared to denervated wildtype muscle (Figure 8b–g). The oxylipins 10‐HDoHE (Figure 8b), 14‐HDoHE (Figure 8e), 12‐HEPE (Figure 8g), and 15‐HEPE (Figure 8h) were higher in cPLA_2_ KO DN when compared to cPLA_2_ KO sham. 16‐HDoHE was higher in muscle from cPLA_2_ KO mice when compared to wildtype mice. Similarly, when we measured oxylipins derived from ω‐6 fatty acids, we found that 5‐HETE was higher in cPLA2 KO sham when compared to wildtype sham (Figure 8i). 12‐HETE, 9,10‐EpOME, and 12,13‐EpOME were higher in cPLA_2_ KO denervated when compared to cPLA_2_ KO sham (Figure 8j–l). 9,10‐EpOME and 12,13‐EpOME were higher in cPLA2 KO DN when compared to wildtype DN (Figure 8k,l). Collectively, these data indicate that cPLA_2_ deletion significantly alters the muscle oxylipin profile that is independent of denervation‐induced muscle loss.

### 3.8 cPLA_2_ deletion alters the transcriptome in sham and denervated muscle

**FIGURE 9:**
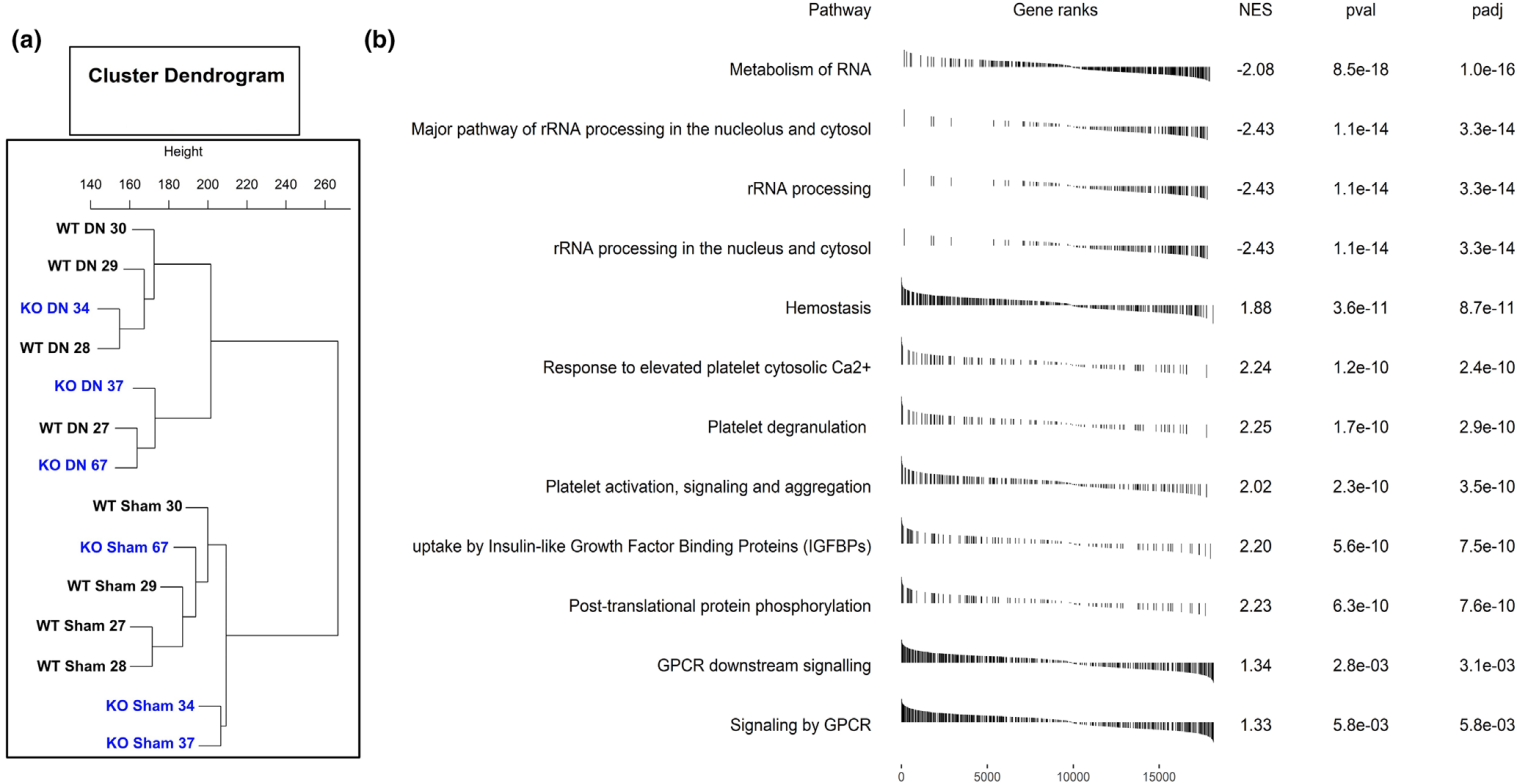
Transcriptomics in sham and denervated wildtype and cPLA_2_ KO mice. (a) Cluster dendrogram of transcriptomics analysis. Hierarchical clustering of all RNA‐seq samples based on global gene expression profiles. Each point corresponds to an individual sample. Sample labels are abbreviated as follows: The first two letters indicate the mouse genotype (WT or KO), followed by the denervation condition (sham or DN), and a trailing number identifying biological replicates within each group. Four biological replicates were included for WT groups and three for DN groups. (b) Gene Set Enrichment Analysis (GSEA) highlighting the top pathways enriched among differentially expressed genes in denervated muscle from wild‐type and cPLA2 knockout (KO) mice. For each pathway, the GSEA enrichment plot displays the distribution of pathway genes across the ranked list of genes ordered by differential expression following cPLA2 deletion. Vertical black bars mark the positions of pathway genes within the ranked list; clustering of these bars toward either end of the plot indicates directional enrichment and altered pathway activity. The normalized enrichment score (NES), nominal *p*‐value, and false discovery rate (adjusted *p*‐value, padj) shown in each panel indicate the statistical significance of enrichment. Positive NES values reflect increased pathway activation in cPLA2 KO muscle, with leading‐edge subsets primarily composed of upregulated genes. An *n* = 3–4 per group was used.

**FIGURE 10:**
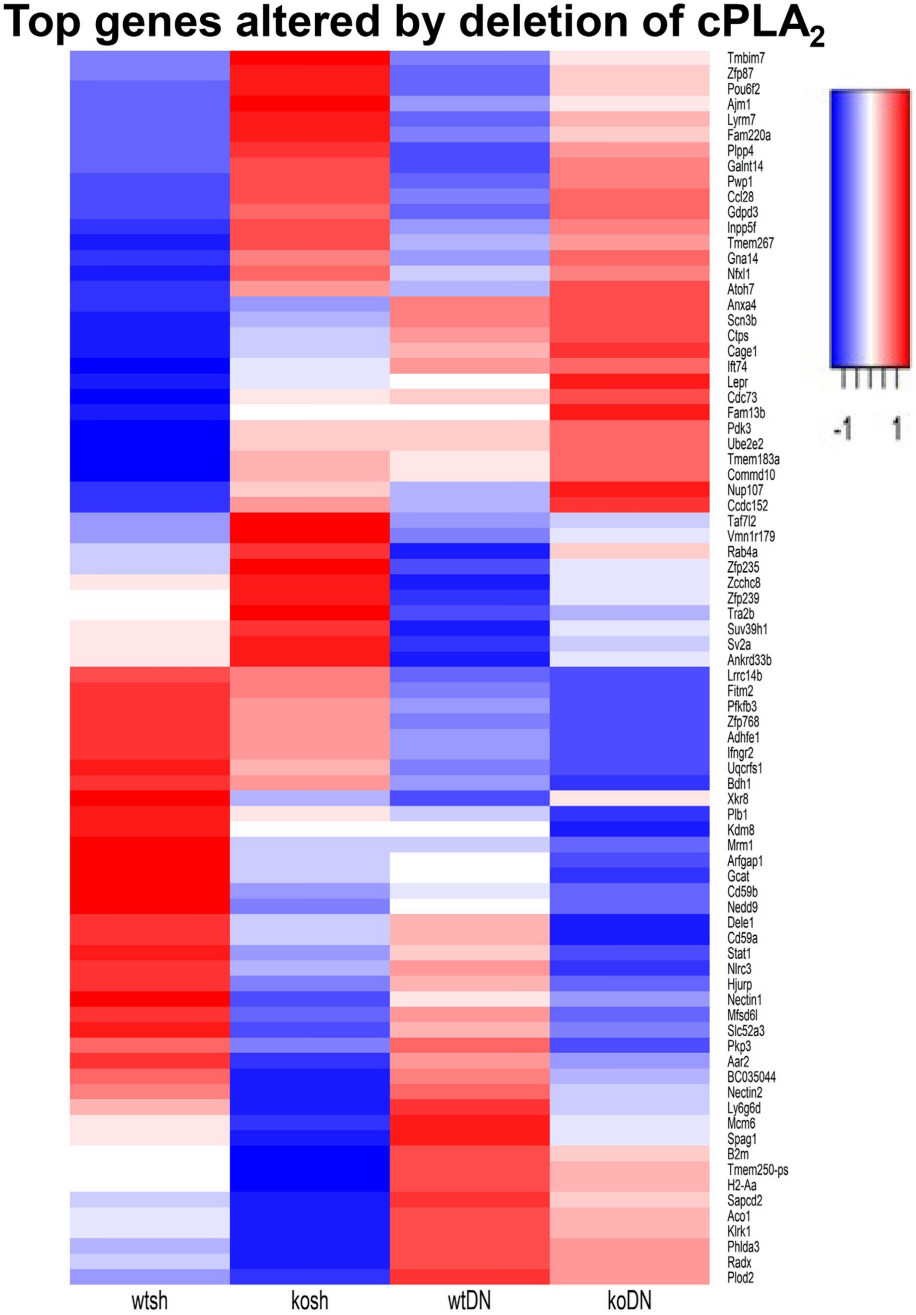
Heatmap of genes showing a significant interaction between genotype (WT vs. cPLA2 KO) and denervation status (sham vs. DN). Strong genotype‐dependent differences in gene expression in sham leg, reflected by pronounced color contrast between (WTsh) and KO (KOsh) first two columns, are diminished in denervation leg (last two columns), highlighting the modulatory effect of cPLA_2_ deletion on denervation‐induced transcriptional changes. An *n* = 3–4 per group was used.

We performed bulk RNA sequencing transcriptomics in gastrocnemius muscle from sham and denervated wildtype and cPLA_2_ KO mice. The cluster dendrogram shows that muscle transcript is altered by denervation (Figure 9a). However, deletion of cPLA_2_ had minimal impact on the muscle transcriptome (Figure 9a) as evidenced by KO and WT clustering together. Figure 9c shows differentially expressed pathways in muscle from WT DN and cPLA_2_ KO DN. These pathways include RNA processing, response to calcium, post‐translational modifications, and G‐protein coupled receptor signaling (Figure 9c). Figure 10 shows the top denervation‐affected genes appear protected by cPLA_2_ deletion. In control mice (Wtsh), the strong color contrast between the first two columns of the heatmap reflects marked differential expression between wildtype (Wtsh) and cPLA_2_ KO muscle (koShm) samples. In KO mice, the difference between WTDN and WT Sham samples is largely diminished (last two columns of the heatmap). For clarity (gene symbol readability), the heatmap is restricted to the 80 genes with the highest interaction effect. Table 1 shows genes that were significantly different when comparing groups.

**TABLE 1:** Summary of changes in genes that were altered in denervation and cPLA_2_ deletion.

Comparison	Gene
WT > cPLA_2_ KO	Lcm14b, Ftm2, Pfkfb3, Zfp768, Adhfe1, Lfngr2, Uqcrfs1, Bdh1, Xkr8, Mrm1, Arfgap1, Gcat, Cd59b, Nedd9, Dele1, Cd59a, Stat1, Nlrc3, and Hjurp
WT<cPLA_2_ KO	Tmbim7, Zfp87, Pou6f2, Ajm1, Lyrm7, Fam220a, Plpp4, Galnt14, Pwp1, Ccl28, Gdpd3, Lnpp5f, Tmem267, Gna14, Nfxl1, Atoh7, Anxa4, Scn3b, Ctps, Cage1, Lft74, Lepr, Cdc73, Fam13b, Pdk3, Ube2e2, Tmem183a, Commd10, Nup107, Ccdc152
DN WT < cPLA_2_ KO DN = SHAM WT	Taf712, Vmn1r179, Rab4a, Zfp235, Zcchc8, Zfp239, Tra2b, Suv39h1, Sv2a, Ankrd33b
DN WT > cPLA_2_ KO DN = SHAM WT	Nectin1, Mfsd6l, Slc52a3, Pkp3, Aar2, Bc035044, Nectin2, Ly6g6d, Mcm6, Spag1, B2m, Tmem250‐ps, H2‐Aa, Sapcd2, Aco1, Klrk1, Phlda3, Radx, Plod2

**FIGURE 11:**
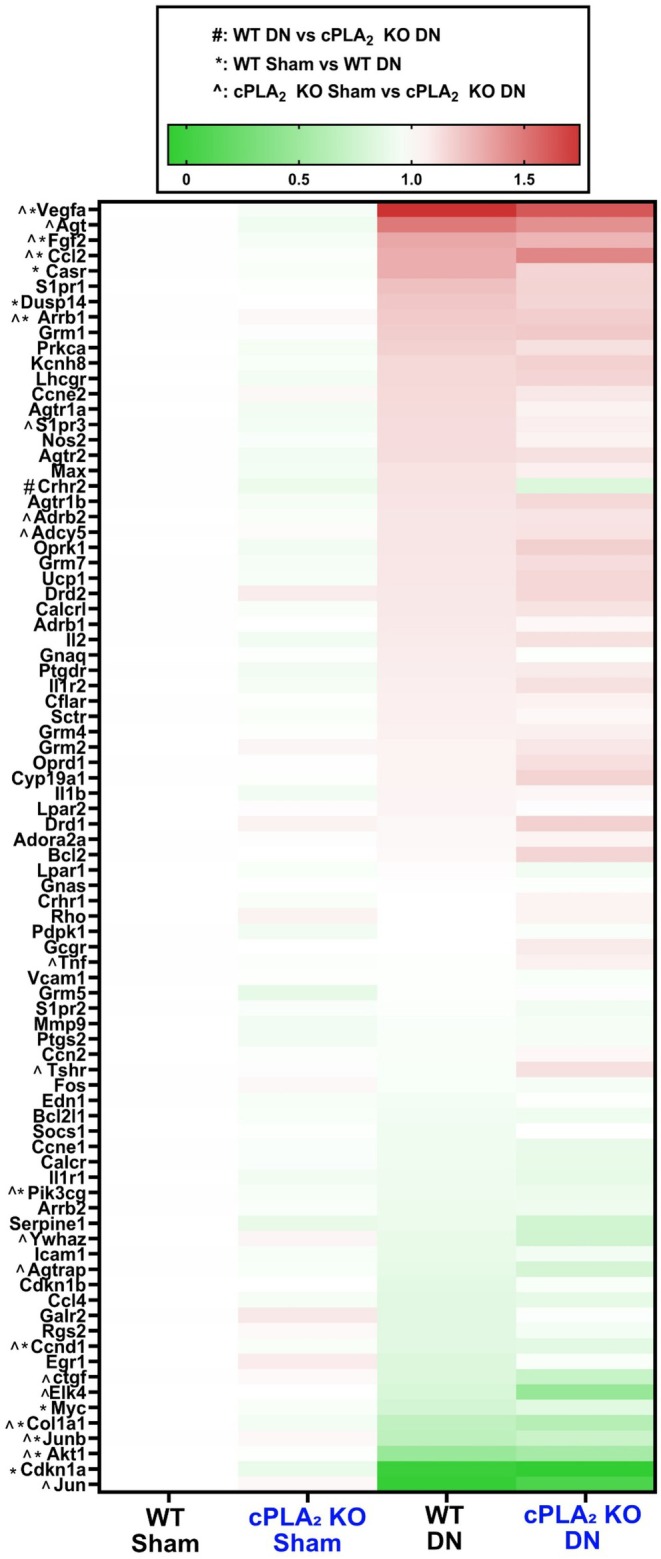
Heatmap showing a G‐protein coupled receptor signaling array. *N* = 6 per group.

Because our transcriptomics data shows G‐protein coupled receptor signaling is differentially expressed in denervated muscle from cPLA_2_ KO and wildtype mice and oxylipins have been shown to work through G coupled protein receptor signaling pathways (Barquissau et al., 2017), we used a targeted G‐protein coupled receptor PCR array to measure mRNA levels in sham and denervated muscle from wildtype and cPLA_2_ KO mice. We measured downstream G‐protein coupled receptor signaling via a PCR array. Figure 11 shows a heat map of genes measured in the array. Table 2 shows genes in the G‐protein coupled receptor signaling PCR array that were significantly different when comparing groups. There was a differential response of genes when comparing wildtype denervated muscle to sham and cPLA_2_ KO denervated muscle to sham, which would indicate that deletion of cPLA_2_ alters G‐protein coupled receptor signaling in denervated muscle. Of note, fewer genes were differentially expressed when comparing cPLA_2_ KO denervated muscle to sham than wildtype denervated muscle to sham (10 genes vs. 25 genes, Table 2).

**TABLE 2:** Summary of changes in the G‐Protein Coupled Receptor Signaling Array.

Comparison	Gene
DN WT > SHAM WT	Galr2, Myc, CCnd1, Rgs2, Icam1, Arrb2, Pik3cg, Cdkn1a, Junb, Akt1, Jun, Col1a1, Agtrap
DN WT < SHAM WT	S1pr1, S1pr3, Agtr1a, Nos2, Dusp14, Arrb1, Casr, Vegfa, Ccl2, Fgf2, Grm1, and Agt
DN in cPLA_2_ KO > SHAM cPLA_2_ KO	Ctgf, Ywhaz
DN in cPLA_2_ KO < SHAM cPLA_2_ KO	Grm5, Bcl2, Drd1, Il1r2, Oprk1, Agtr1b, Lhcgr, Kcnh8

## 4 DISCUSSION

Contrary to our hypothesis and previous results with cPLA_2_ inhibition, the current study found that genetic deletion of cPLA_2_ did not prevent denervation‐mediated muscle atrophy. However, deletion of cPLA_2_ significantly impacted metabolic pathways in skeletal muscle supporting an important role for lipid mediators in muscle. Our data show that proteins involved in oxylipin metabolism and the content of oxylipins were differentially expressed in denervated muscle from wildtype and cPLA_2_ KO mice. In addition, we show that both denervation and deletion of cPLA_2_ change the content of various metabolites. Of note, the metabolite alpha‐hydroxy‐glutarate was lower in muscle from cPLA_2_ KO mice when compared to wildtype mice, which may be consistent with lower HIF‐1α production (Intlekofer et al., 2015). Surprisingly, denervation and deletion of cPLA_2_ seemed to have independent effects on G‐Protein coupled receptor signaling except for the gene *Crhr2*.

We previously showed that the addition of the PLA_2_ inhibitor AACOCF_3_ completely reduces the hydroperoxide signal when added to the fiber bundles in vitro. These data support that state 1 hydroperoxide generation in denervated muscle is PLA_2_ dependent (Pharaoh et al., 2020). Therefore, we hypothesized that genetic deletion of cPLA_2_ would reduce the hydroperoxide signal in denervated muscle. In support of this, we found that genetic deletion of cPLA_2_ lowered state 1 hydroperoxide generation in denervated muscle compared to the amount measured in wildtype muscle after denervation (Figure 6g).

As in our previous work (Pharaoh et al., 2020), we observed that cPLA_2_ and iPLA_2_ were higher in denervated muscle than in innervated muscle; however, in this study, 12/15‐Lox was not altered by denervation. Furthermore, deletion of cPLA_2_ lowered 12/15‐Lox content in muscle when compared to wildtype mice. Intriguingly, deletion of cPLA_2_ did not dramatically alter the muscle oxylipin profile. This may be because of compensatory action from other enzymes such as iPLA_2_, which was higher in denervated muscle in our current study. Another interesting finding in our study was that 12/15‐Lox was lower in cPLA_2_ KO mice when compared to wildtype mice. 12/15‐Lox has previously been implicated as a driver of denervation‐induced muscle atrophy (Bhattacharya et al., 2014). Despite lower protein content of 12/15‐Lox, some 12/15‐Lox metabolites such as 12‐HEPE were higher in the denervated cPLA_2_ KO muscle when compared to wildtype denervated muscle. These data illustrate that the protein content may not be the predominant driver of 12/15‐Lox activity. We also show that GPx4 protein is higher in denervated wildtype muscle but not denervated muscle from cPLA_2_ KO mice. GPx4 overexpression has been shown to mitigate muscle atrophy in aging (Czyżowska et al., 2023), oxidative stress‐induced frailty (Xu et al., 2023) and cancer cachexia (Duggan et al., 2026). Perhaps lower GPx4 content in the denervated cPLA_2_ KO muscle contributed to why we did not observe a protection in denervated muscle.

Based on our prior work (Pharaoh et al., 2020), we hypothesized that genetic deletion of cPLA_2_ like enzymatic inhibition of PLA_2_ would prevent denervation‐induced muscle atrophy. In line with this, when we were phenotyping the cPLA_2_ knockout mice, percent lean mass was higher in cPLA_2_ KO mice compared to wildtype mice. However, the muscle mass and organ mass data do not explain the difference in lean mass in cPLA_2_ KO mice when compared to wildtype mice. Perhaps something not measured like bone density could contribute to the changes observed in lean mass. Contrary to our hypothesis, genetic deletion of cPLA_2_ exacerbated denervation‐induced muscle atrophy despite less mitochondrial hydroperoxide generation. One potential explanation for the differences we observe when treating with the PLA_2_ inhibitor AACOCF_3_ and genetic deletion is that the inhibitor seems to be transient. In our prior in vivo studies using the PLA_2_ inhibitor AACOCF_3_, we observed that AACOCF_3_ would wash out of our fiber bundles, and we could only show that the inhibitor reduced the hydroperoxide signal in denervated muscle if we omitted washing steps prior to the hydroperoxide assay (Pharaoh et al., 2020). In addition, AACOCF_3_ may inhibit other isoforms of PLA_2_. cPLA_2_ is implicated in the production of pro‐resolving lipid mediators and bioactive lysophospholipids, which have many positive effects on cells (Leslie, 2015; Norris et al., 2014; Shindou et al., 2000). There may be systemic effects from cPLA2 deletion that prevent cPLA_2_‐mediated protection against denervation‐atrophy. An example of a systemic effect from cPLA2 deletion could be the upregulation of other PLA_2_ isoforms as there are over 16 different groups of PLA_2_ enzymes that have similar roles to cPLA_2_. Therefore, genetic deletion of cPLA_2_ may remove both positive and negative lipid mediators thus failing to mitigate denervation‐induced muscle atrophy.

In addition to muscle atrophy, denervation alters muscle metabolism (Kostrominova, 2022; Prunonosa Cervera et al., 2021). cPLA_2_ also plays a role in regulating metabolism (Kostrominova, 2022; Prunonosa Cervera et al., 2021). Therefore, we explored whether deletion of cPLA_2_ altered metabolites in denervated muscle. Our data showed that denervation lowered the content of metabolites associated with glycolysis. More work needs to be done to figure out why denervation lowers the content of glycolysis metabolites. Denervation did not alter metabolites from the TCA cycle or mitochondrial respiration. However, α‐hydroxyglutaric acid was significantly lower in muscle from cPLA_2_ KO mice when compared to wildtype mice. α‐hydroxyglutaric acid is a key regulator of HIF‐1α (Intlekofer et al., 2015). The D‐enantiomer impairs HIF‐1α via destabilization, while the L‐enantiomer can stabilize HIF‐1α 1α (Intlekofer et al., 2015). Interestingly, L‐2‐hydroxyglutarate has been shown to protect against ischemic reperfusion injury in cardiac tissue (He et al., 2022). Other work has shown that L‐2‐hydroxyglutarate may sensitize blood cells to ferroptosis (Xi et al., 2023). Therefore, lower levels of α‐hydroxyglutaric acid may contribute to why we did not observe a protection of denervated muscle in cPLA_2_ KO mice.

Prior work shows that denervation may hinder skeletal muscle's ability to use fatty acids as a fuel source (Koonen et al., 2004). Consistent with this literature, we showed that the fatty acid concentration was higher in denervated muscle when compared to innervated control. In addition, our data show that the omega 6:3 ratio is higher in denervated muscle when compared to control. Omega 6 PUFAs such as AA and LA are used to generate pro‐inflammatory oxylipins (Gabbs et al., 2015). In our cPLA_2_ KO model, fatty acid concentration was lower in denervated muscle from cPLA_2_ KO mice compared to denervated muscle from wildtype mice. Further, the omega‐6/omega‐3 ratio was not different in denervated muscle from cPLA_2_ KO mice when compared to innervated muscle from wildtype mice. These data suggest that cPLA_2_ can alter the PUFA concentration in denervated muscle.

Oxylipins derived from n6 PUFAs, such as linoleic acid and arachidonic acid, are typically associated with pro‐inflammatory signaling (Gabbs et al., 2015). Our previous work shows that 12/15‐Lox metabolism of AA and LA may be a source of deleterious oxylipins in denervated muscle (Bhattacharya et al., 2014; Brown et al., 2022). We showed that 12/15‐LOX protein content and oxylipins generated by 12/15‐LOX were higher in denervated muscle compared to control (Pharaoh et al., 2020). We also showed that liproxstatin‐1 treatment, which mitigated denervation‐mediated muscle atrophy, lowered the content of several oxylipins generated by 12/15‐Lox (Brown et al., 2022). We detected a series of oxylipins typically generated from 12/15‐LOX, with 12‐HETE and 14‐HDOHE being most abundant. These oxylipins were higher in cPLA_2_ KO either basally or following denervation. The higher levels of oxylipins generated from 12/15‐LOX were intriguing, considering the enzyme had been significantly downregulated in the muscle from cPLA_2_ KO mice. Specific oxylipins that were higher in denervated muscle from cPLA_2_ KO mice compared to denervated wildtype muscle were 10‐DHoHE, 14‐DHoHE, 12‐HEPE, 9,10‐EpOME, and 12,13‐EpOME. 9,10‐EpOME and 12,13‐EpOME are associated with muscle fatty acid metabolism (Hildreth et al., 2020; Stanford et al., 2018), and 12‐HEPE is associated with glucose uptake in muscle (Leiria et al., 2019). To our knowledge, there are no reports exploring the roles of 10‐DHoHE and 14‐DHoHE in skeletal muscle. Therefore, these results do not explain why denervation‐induced muscle atrophy was not mitigated in cPLA_2_ KO mice.

Our transcriptomics data also showed that ligands associated with G‐protein coupled receptor signaling are differentially expressed in denervated muscle from wildtype and cPLA_2_ KO mice. G‐protein coupled receptors play a critical role in regulating both skeletal muscle hypertrophy and atrophy (Berdeaux & Stewart, 2012). We assessed G‐protein coupled receptor signaling via a qPCR array. The qPCR array showed that connective tissue growth factor (*Ctgf*) was upregulated in denervated muscle from cPLA_2_ KO mice when compared to denervated muscle from wildtype mice. Perhaps the upregulation of *Ctgf* contributes to the altered extracellular matrix signaling observed in our transcriptomics dataset. *Ilr2*, *Bcl2*, *and Agtr1b* are downregulated in denervated muscle from cPLA_2_ KO mice. *Ilr2*, *Bcl2, and Agtr1b* play a role in catabolic signaling in skeletal muscle (Ge et al., 2023; Miao et al., 2021; Takayama et al., 2023). *Agtr1b* is of particular interest in our study as Takayama et al. 2023 showed that suppressed activation of this gene mitigated denervation‐induced muscle atrophy by lowering NFκB and FOXO1 expression (Takayama et al., 2023). Other genes that were differentially expressed in denervated muscle from cPLA_2_ KO mice when compared to wildtype mice were *Lhcgr*, *Ywhaz, Drd1, Kcnh8, Grm5, and Oprk1*.

In summary, we have shown that genetic deletion of cPLA_2_ does not mitigate denervation‐induced muscle atrophy despite lowering muscle hydroperoxide generation. We showed that genetic deletion of cPLA_2_ altered the expression of extracellular matrix and plasma membrane‐related genes. It is not known if the altered expression of extracellular matrix‐related genes leads to altered extracellular matrix composition. Exploring how cPLA_2_ regulates the muscle extracellular matrix could be a worthwhile future direction of study that is relevant for skeletal muscle health.

## AUTHOR CONTRIBUTIONS

**Agnieszka Czyżowska‐Froemling:** Conceptualization; data curation; formal analysis; investigation; methodology; validation. **Hongyang Xu:** Data curation; formal analysis; methodology. **Kylene Harold:** Data curation; formal analysis; methodology; software; visualization. **Jessica Thomason:** Data curation; formal analysis. **Kara Kneuper:** Data curation; formal analysis; methodology. **Elizabeth Duggan:** Data curation; formal analysis. **Atul Pranay:** Data curation; formal analysis; methodology; validation. **Jie Zhu:** Data curation; formal analysis; methodology; validation. **Martin‐Paul Agbaga:** Data curation; formal analysis; methodology; resources. **Constantin Georgescu:** Data curation; formal analysis; methodology; software. **Victoria Tyrrell:** Data curation; formal analysis; resources; software; validation. **Valerie O'Donnell:** Data curation; methodology; resources; supervision; validation. **Ken Humphries:** Resources; supervision; visualization. **Holly Van Remmen:** Conceptualization; data curation; formal analysis; funding acquisition; investigation; methodology; project administration; resources; software; supervision; validation; visualization. **Jacob L. Brown:** Conceptualization; data curation; formal analysis; funding acquisition; investigation; methodology; project administration; resources; software; supervision; validation; visualization.

## CONFLICT OF INTEREST STATEMENT

Authors do not have any conflict of interest.

## ETHICS STATEMENT

All authors adhered to ethical standards in accordance to Physiological Reports.

## Supporting information


Data S1.

## Data Availability

Transcriptomics, lipidomics, and metabolomics datasets can be found: https://doi.org/10.6084/m9.figshare.32125855. All other data will be made available upon request.
